# Preoperative prediction of 5-ALA fluorescence in gliomas: comparison of 7-Tesla magnetic resonance spectroscopic imaging, contrast-enhancement on MRI, and positron emission tomography

**DOI:** 10.1007/s00330-026-12430-w

**Published:** 2026-03-10

**Authors:** Sara Huskic, Philipp Lazen, Cornelius Cadrien, Thomas Roetzer-Pejrimovsky, Barbara Kiesel, Julia Furtner, Johannes Leitner, Anita Kloss-Brandstätter, Lisa Körner, Anna Sophie Berghoff, Matthias Preusser, Günther Grabner, Wolfgang Bogner, Tatjana Traub-Weidinger, Marcus Hacker, Siegfried Trattnig, Karl Rössler, Gilbert Hangel, Georg Widhalm

**Affiliations:** 1 Department of Neurosurgery, Medical University of Vienna, Vienna, Austria; 2 High-field MR Centre, Department of Biomedical Imaging and Image-guided Therapy, Medical University of Vienna, Vienna, Austria; 3 Division of Neuropathology and Neurochemistry, Department of Neurology, Medical University of Vienna, Vienna, Austria; 4 Comprehensive Center for Clinical Neurosciences and Mental Health, Medical University of Vienna, Vienna, Austria; 5 Division of Neuroradiology and Musculoskeletal Radiology, Department of Biomedical Imaging and Image-guided Therapy, Medical University of Vienna, Vienna, Austria; 6 Research Center of Medical Image Analysis and Artificial Intelligence (MIAAI), Danube Private University, Krems, Austria; 7 Department of Medical Engineering, Carinthia University of Applied Sciences, Klagenfurt, Austria; 8 Division of Oncology, Department of Internal Medicine I, Medical University of Vienna, Vienna, Austria; 9 Christian Doppler Laboratory for MR Imaging Biomarkers, Vienna, Austria; 10 Department of Diagnostic and Therapeutic Nuclear Medicine, Clinic Donaustadt, Vienna Health Care Group, Vienna, Austria; 11 Division of Nuclear Medicine, Department of Biomedical Imaging and Image-guided Therapy, Medical University of Vienna, Vienna, Austria

**Keywords:** Brain, Glioma, Magnetic resonance spectroscopy, 5-amino levulinic acid, Fluorescence

## Abstract

**Objectives:**

We investigated whether metabolic ratios derived from ultra-high-field 7-T 3D-FID-CRT-MRSI can predict intraoperatively visible 5-aminolevulinic acid (5-ALA) fluorescence in gliomas and compared their predictive performance to established imaging markers, including contrast enhancement (CE) on MRI and PET tumor-to-normal ratio (TNR).

**Materials and methods:**

We retrospectively analyzed 43 patients with histopathologically confirmed adult-type diffuse gliomas (CNS WHO grades 2–4) who underwent preoperative 7-T MRSI and 5-ALA-guided resection. Group differences between 5-ALA-positive and 5-ALA-negative tumors were tested for 16 metabolic ratios to either total creatine (tCr) or combined N-acetylaspartate and N-acetyl-aspartyl-glutamate (NAA + NAAG; total NAA; tNAA) using non-parametric statistics with Šidák correction. CE-MRI status and PET TNR (subcohort, *n* = 31) were included as reference predictors. We additionally evaluated a subgroup of non-enhancing gliomas (*n* = 27). Receiver operating characteristic (ROC) analysis was performed to determine diagnostic performance.

**Results:**

5-ALA-positive gliomas demonstrated significantly altered metabolic profiles, showing lower mI/tNAA (*p* < 0.001) and higher Gln/tCr, Glx/tCr, Gly/tCr, and GSH/tCr ratios (all *p* < 0.001). These ratios achieved high predictive accuracy for fluorescence (AUC_range_ = 0.79–0.94), comparable or superior to PET TNR (AUC = 0.90) and CE-MRI (AUC = 0.84). In a subcohort of nonenhancing gliomas, Gly/tCr and Gln/tCr showed a high prediction accuracy (AUC = 0.90).

**Conclusion:**

7-T MRSI metabolic ratios can predict intraoperative 5-ALA fluorescence and may serve as an alternative or adjunct to CE-MRI and PET for preoperative patient selection for 5-ALA administration. Finally, these findings could be especially beneficial in non-enhancing gliomas, where CE-MRI offers limited predictive information.

**Key Points:**

***Question*** *Does 7-T MRSI enable preoperative prediction of 5-ALA fluorescence to support patient selection for fluorescence-guided glioma surgery?*

***Findings**** Several 7-T MRSI metabolic ratios (mI/tNAA, Gln/tCr, Glx/tCr, Gly/tCr and GSH/tCr) robustly predicted 5-ALA fluorescence across glioma subtypes, with diagnostic performance comparable to contrast-enhanced MRI and PET*.

***Clinical relevance**** Ultra-high-field 7-T MRSI enables noninvasive preoperative prediction of intraoperative 5-ALA fluorescence in gliomas with performance comparable to PET and contrast-enhanced MRI, supporting surgical planning without the need for contrast agents or radiation exposure*.

**Graphical Abstract:**

**Figure:**
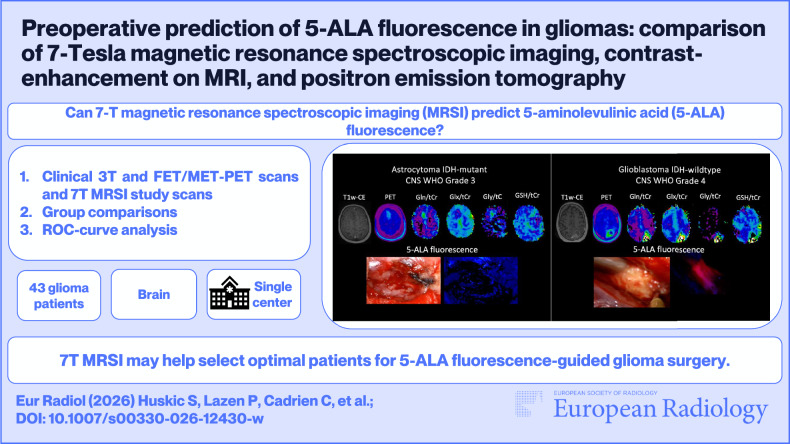

## Introduction

Diffusely infiltrating gliomas constitute the most common primary malignant brain tumors and contribute considerably to cancer mortality [1, 2]. Adult-type diffuse gliomas consist of three distinct tumor types: astrocytomas and oligodendrogliomas with IDH-mutations, and glioblastomas that are IDH-wildtype, with assigned CNS WHO grades 2–4 [1].

Maximal safe resection (MSR) of diffusely infiltrating gliomas is crucial to improve the progression-free and overall survival [3–5]. However, intraoperative detection of residual tumor tissue remains challenging, and incomplete resections are common [3, 6, 7]. To improve intraoperative tumor visualization, fluorescence-guided resection using 5-aminolevulinic acid (5-ALA) was implemented to better distinguish tumor tissue from normal brain tissue and achieve MSR [6, 7]. In tumor cells, 5-ALA is metabolized and accumulated as protoporphyrin IX (PpIX), causing red fluorescence under blue excitation light using a modified neurosurgical microscope [8]. Initially, a disruption of the blood–brain barrier (BBB) was considered a prerequisite for visible 5-ALA fluorescence [9]. Accordingly, 5-ALA fluorescence is typically observed in gliomas showing significant contrast enhancement (CE) on magnetic resonance imaging (MRI) [10]. However, visible 5-ALA fluorescence was also observed in approximately half of gliomas with nonsignificant CE on MRI [11–13]. In these tumors, 5-ALA fluorescence is usually observed only in circumscribed tumor areas frequently corresponding to intratumoral focal malignant transformation (anaplastic foci) [11, 12, 14]. Consequently, 5-ALA enables intraoperative detection of the most malignant tumor areas [11, 12, 15].

In suspected low-grade gliomas (LGG), positron emission tomography (PET) is a powerful tool for detecting intratumoral areas of increased metabolic activity that might represent regions of anaplastic foci [14, 16]. Previous studies found a correlation between visible 5-ALA fluorescence and increased PET tracer uptake [11, 12, 15, 17, 18].

Magnetic resonance spectroscopic imaging (MRSI) is frequently applied as an additional preoperative imaging modality for the detection of intratumoral metabolic activity in gliomas [19, 20]. However, it is unclear whether MRSI can predict 5-ALA fluorescence and therefore be used for selecting patients who could benefit from 5-ALA administration.

Ultra-high-field 7-T MRSI is a noninvasive imaging method that produces high-resolution metabolic maps of various metabolites in the brain, offering metabolic information without the need for contrast agents or radiation exposure [21, 22]. Compared with clinical ≤ 3-T MRSI, which is limited to metabolites such as total N-acetylaspartate (tNAA), total creatine (tCr), and total choline (tCho) [23], 7-T enables higher signal-to-noise ratio (SNR) and improved spectral resolution, allowing depiction of a broader metabolite spectrum and separation of overlapping resonances, such as glutamine (Gln) and glutamate (Glu), as well as myo-inositol (mI) and glycine (Gly) [24–26]. The 3D free induction decay (FID) concentric ring trajectories (CRT) sequence acquires signal immediately after excitation and samples k-space using concentric rings in the xy-plane and phase encoding in the z-direction. This enables efficient 3D coverage with minimal echo-time-related signal loss in clinically feasible scan times [21, 22, 27].

The aim of our study was to investigate the ability of various 7-T metabolites to predict intraoperatively visible 5-ALA fluorescence in a glioma cohort (CNS WHO grades 2–4) and compare the MRSI data to the established imaging methods, including preoperative CE-MRI and PET.

## Materials and methods

All patients provided written informed consent as a part of a prospective 7-T MRSI research protocol approved by the Ethics Committee of the Medical University of Vienna (protocol 1991/2018). The present study is a retrospective analysis of these previously acquired data. No additional imaging or interventions were performed for this analysis.

### Patient recruitment

**Fig. 1:**
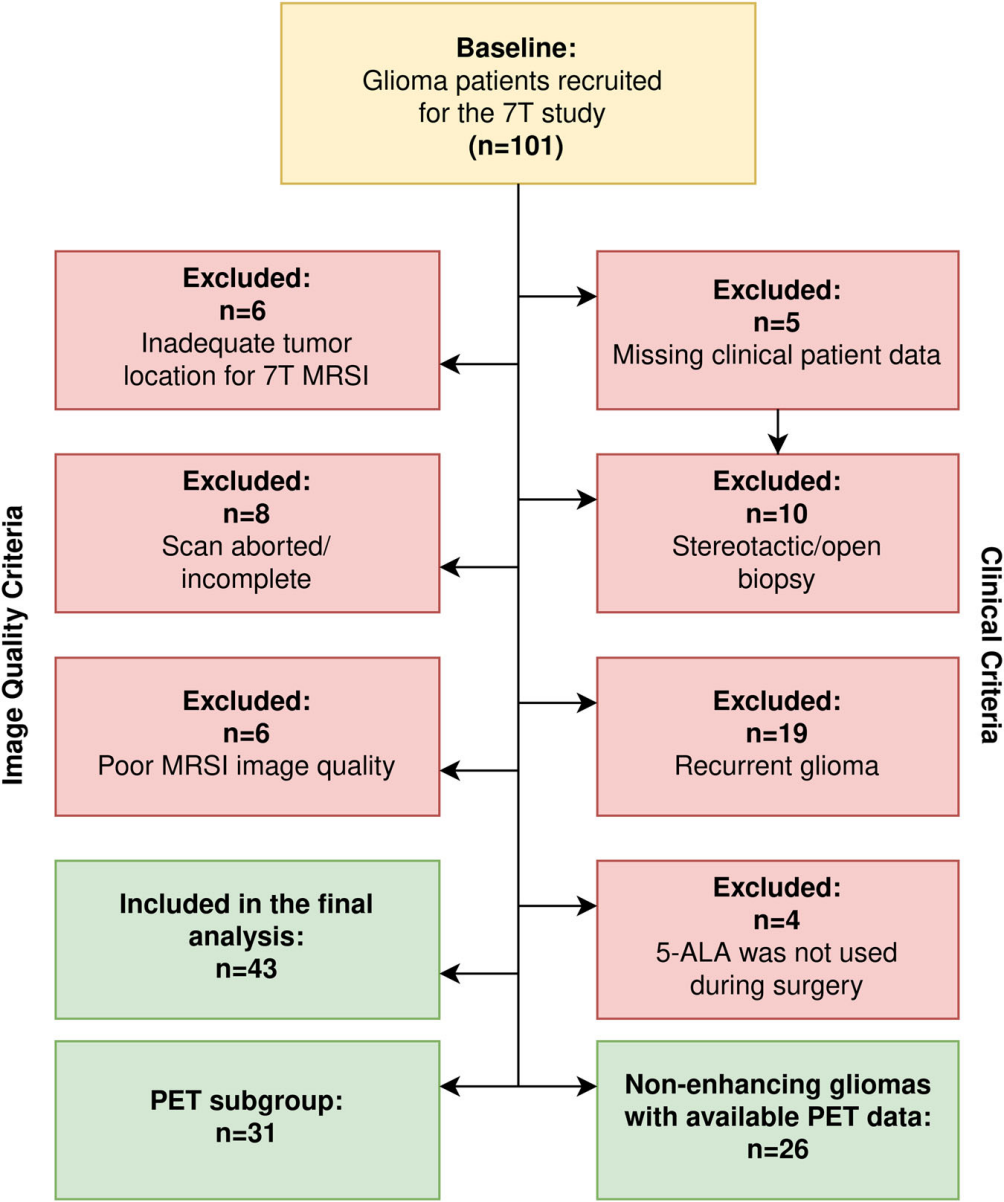
This figure demonstrates the exclusion criteria of our study. Initially, patients were excluded if they had missing clinical data and thus no histological confirmation of the tumor type, stereotactic or open biopsy, recurrent tumors or had surgery without 5-ALA fluorescence. Further, we excluded patients with inadequate location for 7-T MRSI due to field inhomogeneities, poor MRSI quality due to motion artifacts, incomplete or interrupted 7-T MRSI scans. The final cohort consisted of 43 glioma patients, who underwent 5-ALA fluorescence-guided surgery and 7-T MRSI with sufficient image quality, with a subgroup of patients who had an additional PET scan. 5-ALA, 5-aminolevulinic acid; MRSI, Magnetic resonance spectroscopic imaging

We evaluated 101 patients who underwent preoperative 7-T MRSI between 2019 and 2024. Of these, 38 patients (38%) were excluded due to clinical exclusion criteria (missing clinical data, recurrent tumors, biopsy, or absence of 5-ALA administration). Further, 20 cases (32%) were excluded due to 7-T MRSI scan-related issues (inadequate tumor location, aborted/interrupted scans, poor MRSI quality due to motion artifacts). The final cohort consisted of 43 patients with newly diagnosed, histologically confirmed adult-type diffuse gliomas (CNS WHO grades 2–4) and sufficient MRSI data quality. All tumors were classified according to the latest WHO criteria established in 2021 [1], and all CNS WHO grade 2 gliomas were classified as LGG, while CNS WHO grade 3 and CNS WHO grade 4 gliomas were classified as HGG. Exclusion criteria were: stereotactic/open biopsy, recurrent gliomas, and insufficient 7-T MRSI data quality. Details are shown in Fig. 1.

### Preoperative routine clinical imaging

Our routine brain tumor imaging protocol before surgery consisted of 3-T MRI scans. We included the following sequences: T2-weighted, fluid-attenuated inversion recovery (FLAIR), and native and contrast-enhanced T1-weighted [28]. All gliomas were classified based on contrast uptake as either significant CE (nodular or ring-like CE) or nonsignificant CE (none, patchy/faint, or focal CE), as described previously [12]. Furthermore, tumor segmentations using 3-T MRI were performed by neuroradiologists (J.L., J.F.) and served as the regions of interest for MRSI analysis.

### PET data acquisition

We performed preoperative PET scans with amino-acid tracers in patients with nonsignificant CE to detect potential intratumoral increased metabolic activity. Patients received either ^11^C-methionine (MET) or ^18^F-fluoro-ethyl-L-tyrosine (FET) after fasting for 4 h and were scanned using Siemens Biograph mMR PET/MR and Biograph TPTV PET/CT systems (Siemens Healthineers). Tumor standardized uptake values and tumor-to-normal ratio (TNR) were calculated following the PET RANO 1.0 criteria [29].

### 7 Tesla MRSI data acquisition

All patients underwent preoperative MRI scans using a 7-T scanner (Siemens Healthineers) equipped with a 1Tx/32Rx coil (Nova Medical). MRSI data were acquired using a 3D-FID-CRT sequence with a matrix of 64 × 64 × 39 and a nominal isotropic resolution of 3.4 mm, using parameters of Repetition time = 450 ms, acquisition delay = 1.3 ms, and FOV = 220 × 220 × 133 mm³ over 15 min [21]. The data were processed using an in-house MATLAB pipeline. Spectral quantification was done in LCModel [30], using a basis set of 17 metabolites and measured macromolecules [30]. Supplementary Table 1 provides an overview of the processing parameters.

The quality was assured using Cramér-Rao lower bounds < 40%, SNR > 2.5, and full-width at half-maximum < 0.15 ppm. Example spectra are shown in Supplementary Fig. 1. The medians of MRSI ratios in the ROIs were extracted using Python 3.10.12. The metabolic ratios assessed in this study were: tCho/tNAA, Glu/tNAA, Gln/tNAA, Glx/tNA, Gly/tNAA, mI/tNAA, GSH/tNAA, Ser/tNAA, tCho/tCr, Glu/tCr, Gln/tCr, Glx/tCr, Gly/tCr, mI/tCr, GSH/tCr, and Ser/tCr. These ratios were used because of the reliable fitting and the known important role in neuro-oncological imaging [21, 25]. Further, metabolite T1-correction was performed to ensure comparability between sequences using relaxation times as previously reported [27, 31].

### 5-ALA data

Each patient received an oral solution of 5-ALA (20 mg/kg body weight) approximately 3 h before surgery. Intraoperative 5-ALA fluorescence was evaluated in different intratumoral areas using a modified neurosurgical microscope (Kinevo and Pentero, Carl Zeiss Surgical GmbH) and categorized by the neurosurgeons as “positive” and “negative” fluorescence.

### Statistical analysis

Our statistical analysis assessed differences in: sex, age, tumor volume, tumor type, grade, IDH-mutational status, CE-MRI, PET-TNR, MRSI metabolic ratios, and 5-ALA fluorescence status. We applied the Mann–Whitney U test for continuous and the Chi² test for categorical variables. Effect sizes were calculated using Cohen’s d for continuous and Cramér’s V for categorical variables.

To account for multiple comparisons, the Šidák correction set a significance threshold of *p* = 0.002 for all comparisons. To test the fluorescence predictions, we generated receiver operating characteristic (ROC) curves for MRSI ratios, CE-MRI, and, in the PET subgroup, TNR. All statistical analyses were performed using Python 3.1.0.12. Statistical analyses were supervised by an experienced data scientist (A.K.B).

## Results

Our cohort consisted of 43 patients after resection of a newly diagnosed and histologically confirmed adult-type diffuse glioma. Of those, 22 were astrocytoma, IDH-mutant (8 CNS WHO grade 2, 11 CNS WHO grade 3, and 3 CNS WHO grade 4), 10 were oligodendroglioma, IDH-mutant, 1p/19q codeleted (8 CNS WHO grade 2 and 2 CNS WHO grade 3), and 11 CNS WHO grade 4 glioblastoma, IDH-wildtype. Patient data are provided in Table 1.

**Table 1:** Patient characteristics

Patient overview		*n* = 43
Sex	Male: female ratio	22:21 (51%:49%)
Age	Median (range)	45 (26–77)
WHO grade and type	Astrocytoma, IDH-mutant	22/43 (51%)
	CNS WHO Grade 2	8 (17%)
	CNS WHO Grade 3	11 (26%)
	CNS WHO Grade 4	3 (7%)
	Glioblastoma, IDH-wildtype	11/43 (26%)
	CNS WHO Grade 4	11 (26%)
	Oligodendroglioma, IDH-mutant, 1p/19q codeleted	10/43 (23%)
	CNS WHO Grade 2	8 (19%)
	CNS WHO Grade 3	2 (2%)
Localization	Frontal	19 (44%)
	Insular	6 (14%)
	Parietal	8 (19%)
	Central	2 (5%)
	Temporal	5 (11%)
	Occipital	3 (7%)
5-ALA fluorescence	Positive	20 (47%)
	Negative	23 (53%)
Contrast enhancement	Nonsignificant	29/43 (67%)
	None	14 (33%)
	Focal	4 (9%)
	Patchy/faint	11 (25%)
	Significant	14/43 (33%)
	Ring-like	14 (33%)
	Nodular	0 (0%)
PET imaging	PET subgroup	31/43 (72%)
	Tumor-to-normal ratio (TNR)	1.87 (0.15–4.8)

*5-ALA* 5-aminolevulinic acid

### Patient characteristics

The median age was 45 with a range from 26 to 77 years and a female to male ratio of 1.1:1. The most common tumor localization was frontal (*n* = 19), followed by parietal (*n* = 8) and insular (*n* = 6). In the 43 patients, 29 (67%) tumors showed nonsignificant CE (patchy, faint, or none), while 14 tumors (33%) showed significant (ring-like) CE. PET scans were conducted in a subcohort of 31 (72%) patients. This cohort consisted of 18 astrocytomas IDH-mutant (6 CNS WHO grade 2, 11 CNS WHO grade 3, and 1 CNS WHO grade 4), 10 oligodendroglioma IDH-mutant, 1p/19q codeleted (8 CNS WHO grade 2 and 2 CNS WHO grade 3), and 3 CNS WHO grade 4 glioblastoma, IDH-wildtype. The median PET TNR was 1.87 with a range from 0.15 to 4.88. For more details, see Table 1.

### 5-ALA fluorescence groups

During surgery, 20 gliomas (47%) showed visible 5-ALA fluorescence (5-ALA positive group) and 23 tumors (53%) did not (5-ALA-negative group). The groups did not significantly differ in age, sex, or tumor volume between groups. With regard to histopathological tumor diagnosis, the rate of HGG was significantly higher in the 5-ALA positive group (*p* < 0.001). We did not find a significant difference in the IDH-mutational status and glioma type between fluorescing and non-fluorescing gliomas. Details are shown in Table 2.

**Table 2:** Differences in specific parameters between the 5-ALA-negative and positive groups

Full cohort		5-ALA negative	5-ALA positive	*p*-value	d
General	Patients	23	20	-	-
	Age	35 (26–77)	50.5 (29–72)	0.003	0.54
	Sex (m:f)	8:14	15:6	0.046	0.30
	Volume (cm³)	53.71 (1.73–134)	41.56 (0.9–150)	0.567	−0.10
Histology	Oligodendroglioma	9	1	**< 0.001**	**0.58**
	Astrocytoma	13	9	**< 0.001**	**0.58**
	Glioblastoma	1	10	**< 0.001**	**0.58**
	Grade (low:high)	15:8	1:19	**< 0.001**	**0.57**
	IDH (mut:WT)	22:1	10:10	0.002	0.47
Clinical imaging	CE-MRI (0:1)	23:0	6:14	**< 0.001**	**0.83**
	PET TNR (median)*	1.61 (0.15–3.6)	3.69 (1.60–4.88)	**< 0.001**	**0.74**

Significant differences were present with regard to sex, tumor grade, contrast enhancement (CE) on MRI, and PET tumor-to-normal brain ratio (TNR) in the PET subgroup.

Bold values indicate statistically significant results after Šidák correction for multiple comparisons. The significance threshold was set to *p* = 0.002

* The PET TNR was calculated for the PET subgroup (*n* = 31)

*WT* wild-type

### 5-ALA fluorescence and established imaging parameters

Based on preoperative 3-T MRI, significant CE was detected in 14 (52%) of the 27 HGG (CNS WHO grades 3 and 4) and in none of the 16 LGG (CNS WHO grade 2). The presence of significant CE was more common in the 5-ALA positive group (*p* < 0.001). Of the 31 patients in the PET subgroup, 11 tumors (35%) were in the 5-ALA positive group and 20 (64%) in the 5-ALA negative group. The PET TNRs were significantly higher in the 5-ALA-positive (TNR_mean_ = 3.27) compared to the 5-ALA-negative group (TNR_mean_ = 1.72; *p* < 0.001).

### 5-ALA and 7-T MRSI ratios

**Fig. 2:**
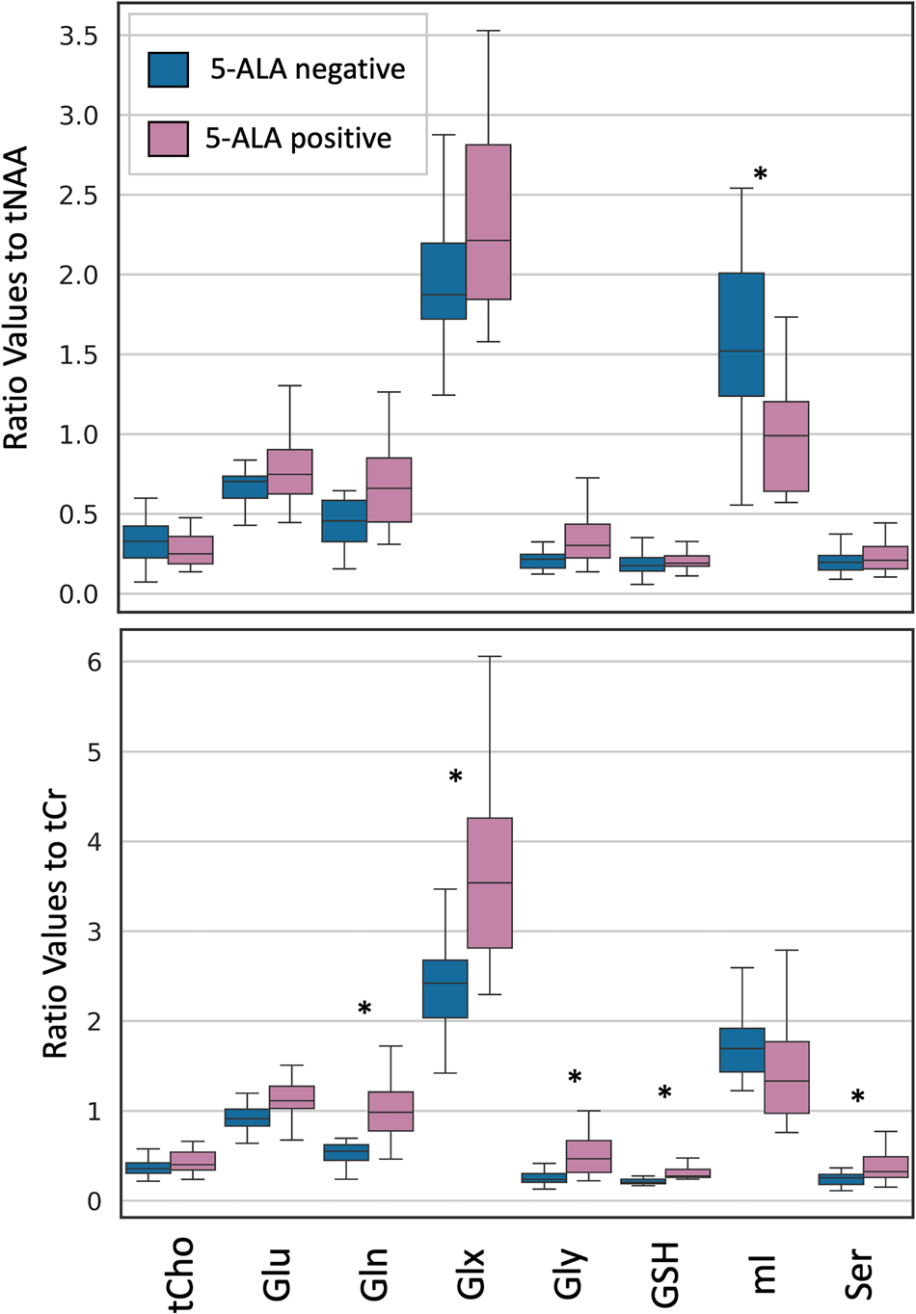
Boxplots showing distributions of median values of 7-T MRSI metabolic ratios in the 5-ALA-negative (blue) and 5-ALA-positive (red) groups. Differences were tested using Mann–Whitney U test. Using Šidák correction for multiple comparisons, the significance threshold was set to *p* = 0.002. Significant differences were found in mI/tNAA, Gln/tCr, Glx/tCr, Gly/tCr, and GSH/tCr. * *p* < 0.001. 5-ALA, 5-aminolevulinic acid; MRSI, Magnetic resonance spectroscopic imaging; tCho, total choline; tCr, total creatine; Glu, glutamate; Gln, glutamine; Glx, combined glutamine and glutamate signal; Gly, glycine; GSH glutathione; mI, myo-inositol; Ser, Serine; tNAA, total N-acetylaspartate + N-acetyl-aspartyl-glutamate

**Fig. 3:**
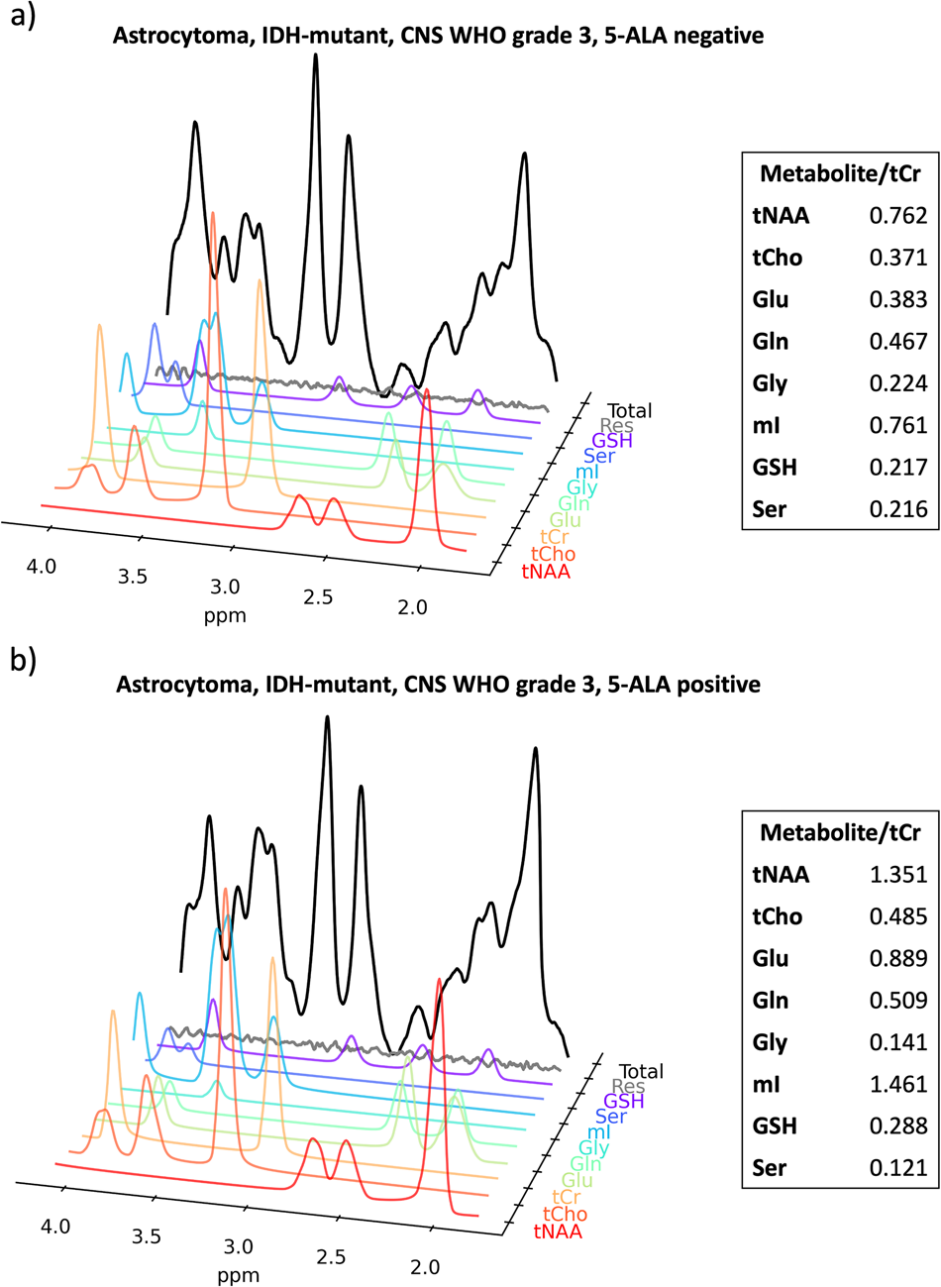
Representative averaged spectra from all tumor voxels within the tumor segmentation (region of interest) of **a** an astrocytoma, IDH-mutant, CNS WHO grade 3, 5-ALA negative patient, and **b** an astrocytoma, IDH-mutant, CNS WHO grade 3, 5-ALA positive patient. Spectral data were phase-corrected prior to averaging across tumor voxels within each patient. The averaged spectra were fitted using LCModel. The figure displays the averaged spectra, the residual after fitting, and the individual fitted contributions of evaluated metabolites, including ratios to total creatine (tCr). In this representative example, the 5-ALA positive patient exhibits higher metabolite-to-tCr ratios for tCho, Glu, Gln, mI, and GSH

**Fig. 4:**
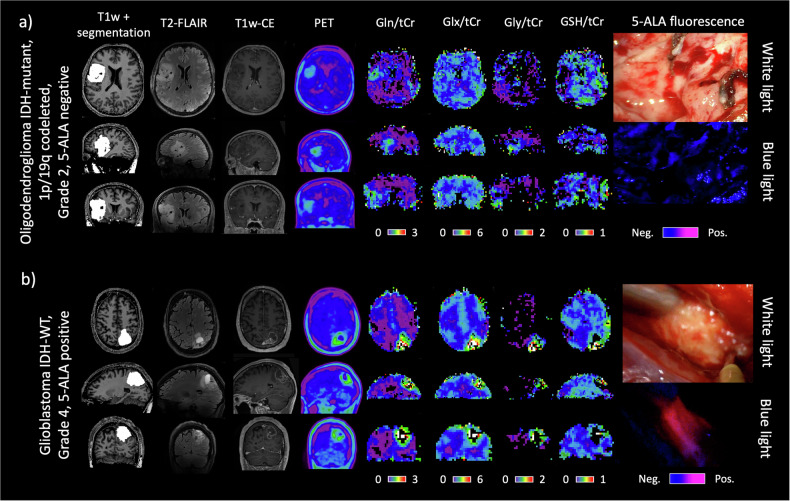
Illustrative examples of preoperative imaging parameters, PET scan, different 7-T MRSI metabolic maps, and corresponding intraoperative images under conventional white-light and 5-ALA fluorescence microscopy in two glioma patients of the 5-ALA-negative and positive groups. **a** The first patient (male, 61 years old) with an IDH-mutant astrocytoma, WHO grade 2–3, shows a relatively homogenous T2/FLAIR hyperintense lesion in the right frontal lobe. The lesion shows nonsignificant CE on MRI (no CE) and, according to PET imaging, an increased tracer uptake is present (TNR = 3.00). According to 7-T MRSI metabolic maps, partial Gln/tCr (0.62), no Glx/tCr (1.41) hotspot, no Gly/tCr hotspot (0.41), and a GSH/tCr (0.19) coldspot are present. The tumor tissue is shown under intraoperative white-light microscopy and shows no visible 5-ALA fluorescence in the entire tumor area. **b** The second patient (male, 52 years old) with an IDH-wildtype glioblastoma, WHO grade 4, shows a relatively heterogeneous T2/FLAIR hyperintense lesion in the left occipital lobe. The lesion shows significant CE on MRI (ring-like CE) and, according to PET imaging, a markedly increased tracer uptake is present (TNR = 3.63). According to 7-T MRSI metabolic maps, distinct Gln/tCr (0.63), Gly/tCr (0.28), Glx/tCr (1.23), and GSH/tCr (0.31) “hotspots” are detected. The tumor tissue is shown under intraoperative white-light microscopy, and visible 5-ALA fluorescence is detected under fluorescence microscopy. 5-ALA, 5-aminolevulinic acid; MRSI, Magnetic resonance spectroscopic imaging; tCho, total choline; tCr, total creatine; Glu, glutamate; Gln, glutamine; Glx, combined glutamine and glutamate signal; Gly, glycine; GSH glutathione; mI, myo-inositol; Ser, Serine; tNAA, total N-acetylaspartate + N-acetyl-aspartyl-glutamate

According to our data, we found significantly lower median values of mI/tNAA (1.52 vs 0.09; *p* < 0.001) and higher medians of Gln/tCr (0.55 vs 0.98; *p* < 0.001), Glx/tCr (2.42 vs 3.54; *p* < 0.001), Gly/tCr (0.23 vs 0.47; *p* < 0.001), and GSH/tCr (0.20 vs 0.27; *p* < 0.001) in the 5-ALA-positive group compared to the 5-ALA-negative group. Details are shown in Fig. 2 and Table 3. Figures 3 and 4 show representative 7-T MRSI spectra and maps from the 5-ALA-positive and negative groups.

**Table 3:** Differences in 7-T MRSI metabolic ratios in the 5-ALA-negative and positive groups

		Metabolic ratio differences between the 5-ALA groups							
	/tNAA	Group medians		*p*-value	d	/tCr	Group medians	*p*-value	d
Metabolic ratio	tCho	0.33 vs 0.25	0.401		0.01	tCho	0.36 vs 0.40	0.110	0.47
	Glu	0.70 vs 0.75	0.141		0.52	Glu	0.91 vs 1.11	0.002	0.93
	Gln	0.46 vs 0.66	0.007		0.88	Gln	**0.55 vs 0.98**	**< 0.001**	**1.96**
	Glx	1.87 vs 2.21	0.033		0.80	Glx	**2.42 vs 3.54**	**< 0.001**	**1.62**
	mI	**1.52 vs 0.99**	**< 0.001**		**−1.04**	mI	1.69 vs 1.33	0.013	−0.74
	Gly	0.22 vs 0.30	0.005		1.00	Gly	**0.23 vs 0.47**	**< 0.001**	**1.30**
	GSH	0.17 vs 0.19	0.267		0.38	GSH	**0.20 vs 0.27**	**< 0.001**	**1.30**
	Ser	0.20 vs 0.21	0.429		0.50	Ser	0.25 vs 0.32	0.006	0.69

The table demonstrates the differences in the median metabolic ratio values between the 5-ALA-negative and the 5-ALA-positive groups, with significant differences between: mI/tNAA, Gln/tCr, Glx/tCr, Gly/tCr, and GSH/tCr. Bold values indicate statistically significant results after Šidák correction for multiple comparisons. The significance threshold was set to *p* = 0.002. 5-*ALA* 5-aminolevulinic acid, *MRSI* Magnetic resonance spectroscopic imaging, *tCho* total choline, *tCr* total creatine, *Glu* glutamate, *Gln* glutamine, *Glx* combined glutamine and glutamate signal, *Gly* glycine, *GSH* glutathione, *mI* myo-inositol, *Ser* Serine, *tNAA* total N-acetylaspartate + N-acetyl-aspartyl-glutamate

### 5-ALA fluorescence prediction using 7-T MRSI ratios and established imaging techniques

#### Entire cohort

**Fig. 5:**
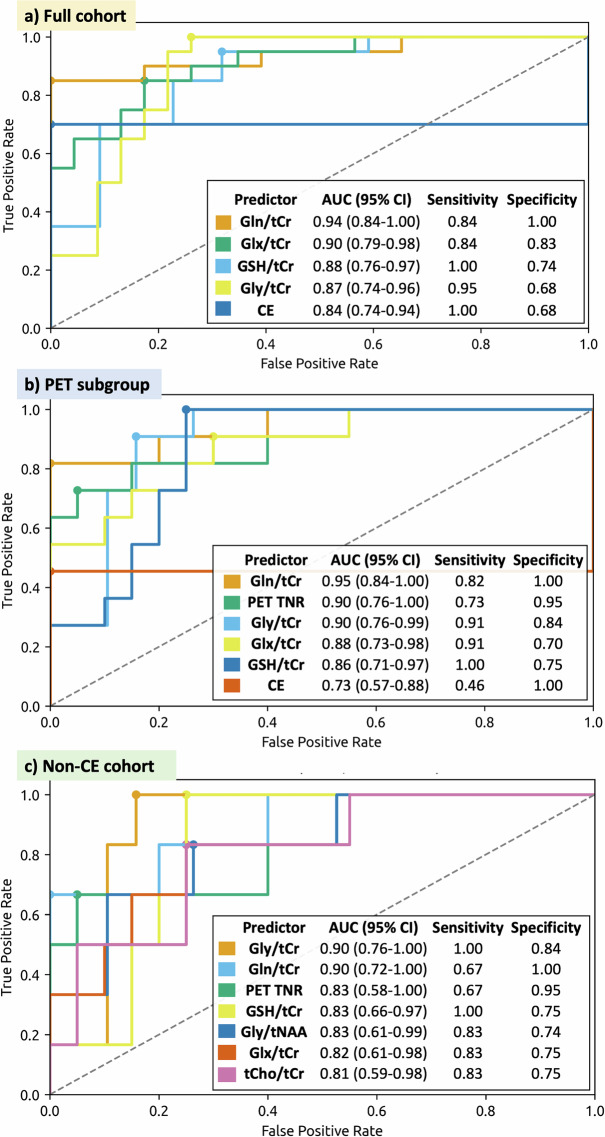
Receiver operating curves (ROC) with their respective area under the curve (AUC) values with confidence intervals (CIs), sensitivity and specificity showing the ability of individual 7-T MRSI metabolic ratios (Gln/tCr, Glx/tCr, Gly/tCr, GSH/tCr, and mI/tNAA) to predict the presence of 5-ALA fluorescence compared to established imaging parameters (CE on MRI and PET TNR). The predictions were tested on **a** the full cohort, **b** the PET subgroup, and **c** the non-enhancing glioma subgroup. The highest predictor was Gln/tCr (AUC = 0.94), followed by Glx/tCr (AUC = 0.91) and GSH/tCr (AUC = 0.88). Contrast enhancement on MRI had an AUC of 0.85, and in the PET TNR in the PET subgroup had an AUC of 0.90. 5-ALA, 5-aminolevulinic acid; MRSI, Magnetic resonance spectroscopic imaging; tCho, total choline; tCr, total creatine; Glu, glutamate; Gln, glutamine; Glx, combined glutamine and glutamate signal; Gly, glycine; GSH glutathione; mI, myo-inositol; Ser, Serine; tNAA, total N-acetylaspartate + N-acetyl-aspartyl-glutamate

In the full cohort (*n* = 43), the MRSI ratios including mI/tNAA, Gln/tCr, Glx/tCr, Gly/tCr, and GSH/tCr predicted the presence of 5-ALA fluorescence with AUC scores of 0.80, 0.94, 0.91, 0.87, and 0.88, respectively. The presence of CE predicted the 5-ALA fluorescence with an AUC of 0.84. For details, see Fig. 5a.

#### PET subgroup

Finally, to enable direct comparison of PET and MRSI, we used a PET subgroup (*n* = 31) in patients with available PET data. PET TNR showed an AUC of 0.90. Additionally, in this subgroup, mI/tNAA, Gln/tCr, Glx/tCr, Gly/tCr, and GSH/tCr showed AUC scores of 0.79, 0.95, 0.88, 0.90, and 0.86, respectively. Further, CE-MRI showed an AUC of 0.73 for 5-ALA fluorescence prediction. Details are provided in Fig. 5b.

#### Non-enhancing glioma subgroup

A subgroup of patients without significant CE (*n* = 26) with available PET scans was additionally analyzed to assess the added value of 7-T MRSI in non-enhancing gliomas. In this subgroup, Gly/tCr, Gln/tCr, GSH/tCr, Gly/tNAA, Glx/tCr, and tCho/tCr showed an AUC > 0.80, with Gly/tCr and Gln/tCr having the highest AUCs (0.90). In this subgroup, PET TNR showed an AUC of 0.83. Details are seen in Fig. 5c.

## Discussion

Generally, the pattern of preoperative CE and the PET metabolic activity are established techniques in the routine clinical practice to predict visible 5-ALA fluorescence prior to glioma surgery [15, 32, 33]. This is the first analysis of the ability of 7-T MRSI ratios to predict visible 5-ALA fluorescence in gliomas of different grades and types. We compared our data to the established imaging methods CE-MRI and PET.

### 5-ALA fluorescence and CE on preoperative MRI

Disruption of the BBB results in the occurrence of significant CE on preoperative MRI. This is a major factor in the presence of visible 5-ALA fluorescence during surgery [13]. We found that significant CE on preoperative MRI was significantly more common in gliomas with visible 5-ALA fluorescence. Similarly, Ewelt et al observed visible 5-ALA fluorescence in the majority of gliomas with detectable CE (13 of 14 gliomas; 93%) [15]. Moreover, Jaber et al analyzed 166 gliomas lacking typical glioblastoma imaging features and found that most enhancing gliomas (78%) showed visible 5-ALA fluorescence, whereas most non-enhancing tumors did not (84%) [34]. Overall, both our findings and the literature confirm a strong correlation between CE and 5-ALA fluorescence.

### 5-ALA fluorescence and PET imaging

Since 5-ALA fluorescence is also found in approximately half of gliomas without significant CE [11, 12, 32], fluorescence is not limited to gliomas with a clear BBB disruption [13]. In these cases, PET imaging was identified as a powerful tool to predict 5-ALA fluorescence [32]. Accordingly, we found that patients with significantly higher PET-TNR values showed 5-ALA fluorescence more frequently. Similarly, Stockhammer et al [17] and Widhalm et al [12] found significant differences in PET-TNR between 5-ALA-positive and negative glioma patients. Further, Jaber et al showed that in gliomas lacking typical glioblastoma imaging features, visible 5-ALA fluorescence occurs in ~46% if the FET-PET uptake ratio exceeds 1.85 and the tumor volume is > 10.6 cm^3^ of volume [32]. These independent observations highlight the significant association between 5-ALA fluorescence and PET in gliomas.

### 5-ALA fluorescence and 7-T MRSI ratios

Using our 3D-FID-CRT-MRSI sequence, we are able to produce high-resolution 3D metabolic maps and analyze a wide range of metabolites [21, 22, 26]. To our knowledge, this is the first study that analyzed the potential of MRSI to predict the presence of 5-ALA fluorescence gliomas. We found significant differences in the median ratios of: mI/tNAA, Gln/tCr, Glx/tCr, Gly/tCr, and GSH/tCr between 5-ALA positive and negative groups.

Patients with lower mI/tNAA median values were more likely to show 5-ALA fluorescence. Previous magnetic resonance spectroscopy (MRS) studies have linked decreased mI levels to poorer prognosis and higher tumor grade, suggesting that mI may have antitumor effects [35, 36]. In HGG, usually demonstrating visible 5-ALA fluorescence, the disruption of the BBB results in reduced mI levels [37].

We provide the first in vivo evidence suggesting that higher Gln/tCr levels are more likely to exhibit 5-ALA fluorescence. In gliomas, Gln, a part of the Gln-Glu cycle, serves as a critical source of nitrogen, fueling cell growth and survival [38]. Tumors with increased Gln storage or catabolism are often associated with increased metabolic activity and rapid tumor progression [38]. Kim et al found that the downregulation of the glutaminase 2 gene results in impaired 5-ALA metabolism and PpIX accumulation, suggesting a potential mechanism to explain different fluorescence patterns [39]. Gln is also converted to glutamate (Glu), the most abundant excitatory neurotransmitter [40]. In gliomas, extracellular Glu plays an important role in seizure induction and aids the infiltration of tumor cells into the surrounding tissue [41]. In 1.5–3-T MRSI, the separation of the Gln signal from Glu is unreliable and thus usually their sum (Glx) is investigated [42]. In our study, Glx/tCr was found to be significantly increased in the 5-ALA-positive group.

Moreover, gliomas with visible 5-ALA fluorescence showed increased median Gly/tCr ratios. Endogenous 5-ALA depends on Gly and succinyl-CoA levels, which condense to form 5-ALA [13]. Elevated Gly levels have been linked to high proliferation rates and tumor aggressiveness, potentially connected to the one-carbon metabolism pathway [43].

Finally, there was an increase in the median GSH/tCr ratios in fluorescing gliomas. GSH is a predominant antioxidant in the human body, and its levels have been shown to be elevated in gliomas [44]. Its primary role is associated with treatment resistance, as it protects tumor cells from chemo- and radiotherapy and supports tumor growth in hypoxic conditions [40].

### Predicting 5-ALA fluorescence

In our ROC analysis, we found that the presence of CE and the PET TNR are powerful predictors for 5-ALA fluorescence (AUC = 0.84 and AUC = 0.90, respectively). Previously, Jaber et al demonstrated a slightly lower overall diagnostic accuracy (69%) for FET-PET as a predictor of 5-ALA fluorescence, whereas the presence of CE showed a higher diagnostic accuracy (81%) compared to our findings [32]. Gln/tCr, Glx/tCr, Gly/tCr, GSH/tCr, and mI/tNAA all predicted 5-ALA fluorescence with higher accuracy. The presence of 5-ALA fluorescence in non-enhancing gliomas highlights the limitations of CE-MRI for prediction. In our non-CE subgroup, several MRSI ratios predicted 5-ALA fluorescence, suggesting that MRSI could detect metabolic alterations associated with fluorescence that are not reflected by CE.

### Clinical relevance and outlook

The main clinical application of our findings is that 7-T MRSI may serve as an alternative or in combination with CE-MRI and/or PET to preoperatively select patients with a high likelihood of visible 5-ALA fluorescence during surgery. The alternative use of MRSI for preoperative prediction of 5-ALA fluorescence could benefit centers without access to PET imaging, patients with contraindications to gadolinium, while avoiding radiation exposure [45]. Moreover, MRSI can visualize different metabolic maps, offering insights into multiple metabolic and tumor biological pathways compared to PET imaging. After identifying the most promising MRSI ratios with the assistance of ultra-high-field 7-T MRSI, we aim to translate our findings to routine clinical practice. Methods such as spectral editing MRS are promising for imaging specific metabolites on clinical field strengths [46]. Future prospective studies should topographically correlate MRSI hotspots with intraoperative 5-ALA fluorescence, CE-MRI, and PET hotspots using neuronavigation. Furthermore, recent acceleration and reconstruction advances are improving the stability and speed of MRSI acquisitions, thus minimizing scan interruptions and motion artifacts [47].

### Limitations

A main limitation of this study was the relatively small cohort size, which limits statistical robustness. Further, 3D-FID-CRT-MRSI sequence has restricted spatial coverage. Brain regions located near air-tissue interfaces are prone to magnetic field inhomogeneities, which compromise imaging reliability. The use of metabolic ratios rather than absolute metabolite concentrations provides only indirect estimates of metabolite levels, although T1-corrected ratios have been shown to be reproducible across centers and field strengths [27, 31]. Finally, the predictive models were not validated on an independent test cohort with reported values requiring larger, external cohorts for validation.

## Conclusions

This study was the first to investigate the ability of various 7-T MRSI ratios in predicting intraoperative 5-ALA fluorescence in a series of 43 gliomas of different types and grades compared to established imaging methods. We found that several 7-T MRSI ratios, including mI/tCr, Gln/tCr, Glx/tCr, Gly/tCr, and GSH/tCr, could distinguish between fluorescing and non-fluorescing gliomas. Consequently, these findings suggest that 7-T MRSI may help in preoperative patient selection for 5-ALA administration and seem to be especially beneficial in non-enhancing glioma cases, where CE-MRI offers limited predictive information.

## Supplementary information

ELECTRONIC SUPPLEMENTARY MATERIAL

## Abbreviations

1p/19q: Co-deletion of chromosome arms 1p and 19q
3 T: 3 Tesla (clinical MRI field strength)
5-ALA: 5-Aminolevulinic acid
7 T: 7 Tesla (ultra-high-field MRI)
AUC: Area under the (ROC) curve
BBB: Blood–brain barrier
CE: Contrast enhancement/contrast-enhancing
CE-MRI: Contrast-enhanced magnetic resonance imaging
CNS WHO: World Health Organization classification of central nervous system tumors
CRT: Concentric ring trajectories
FET: ¹⁸F-fluoro-ethyl-L-tyrosine
FID: Free induction decay
FLAIR: Fluid-attenuated inversion recovery
Gln: Glutamine
Glu: Glutamate
Glx: Combined glutamine + glutamate signal
Gly: Glycine
GSH: Glutathione
HGG: High-grade glioma
IDH: Isocitrate dehydrogenase
LGG: Low-grade glioma
MET: ¹¹C-methionine
mI: Myo-inositol
MRS: Magnetic resonance spectroscopy
MRSI: Magnetic resonance spectroscopic imaging
MSR: Maximal safe resection
PpIX: Protoporphyrin IX
ROC: Receiver operating characteristic
Ser: Serine
SNR: Signal-to-noise ratio
tCho: Total choline
tCr: Total creatine
tNAA: Total N-acetylaspartate + N-acetyl-aspartyl-glutamate (total NAA)
T1: Longitudinal relaxation time (also in T1-weighted imaging)
T2: Transverse relaxation time (also in T2-weighted imaging)
TNR: Tumor-to-normal ratio
