# Supplementary material for: Preoperative prediction of 5-ALA fluorescence in gliomas: comparison of 7-Tesla magnetic resonance spectroscopic imaging, contrast-enhancement on MRI, and positron emission tomography
Source: Eur Radiol. 2026 Mar 10;36(7):5469–81. doi: 10.1007/s00330-026-12430-w (PMC13282335; doi:10.1007/s00330-026-12430-w)
Supplement: Supplementary file 1 — ELECTRONIC SUPPLEMENTARY MATERIAL [file 330_2026_12430_MOESM1_ESM.pdf]

**Preoperative prediction of 5-aminoloevulinic acid fluorescence in gliomas: comparison of 7 Tesla magnetic resonance spectroscopic imaging, contrast-enhancement on MRI, and positron emission tomography**

**ELECTRONIC SUPPLEMENTARY MATERIAL**

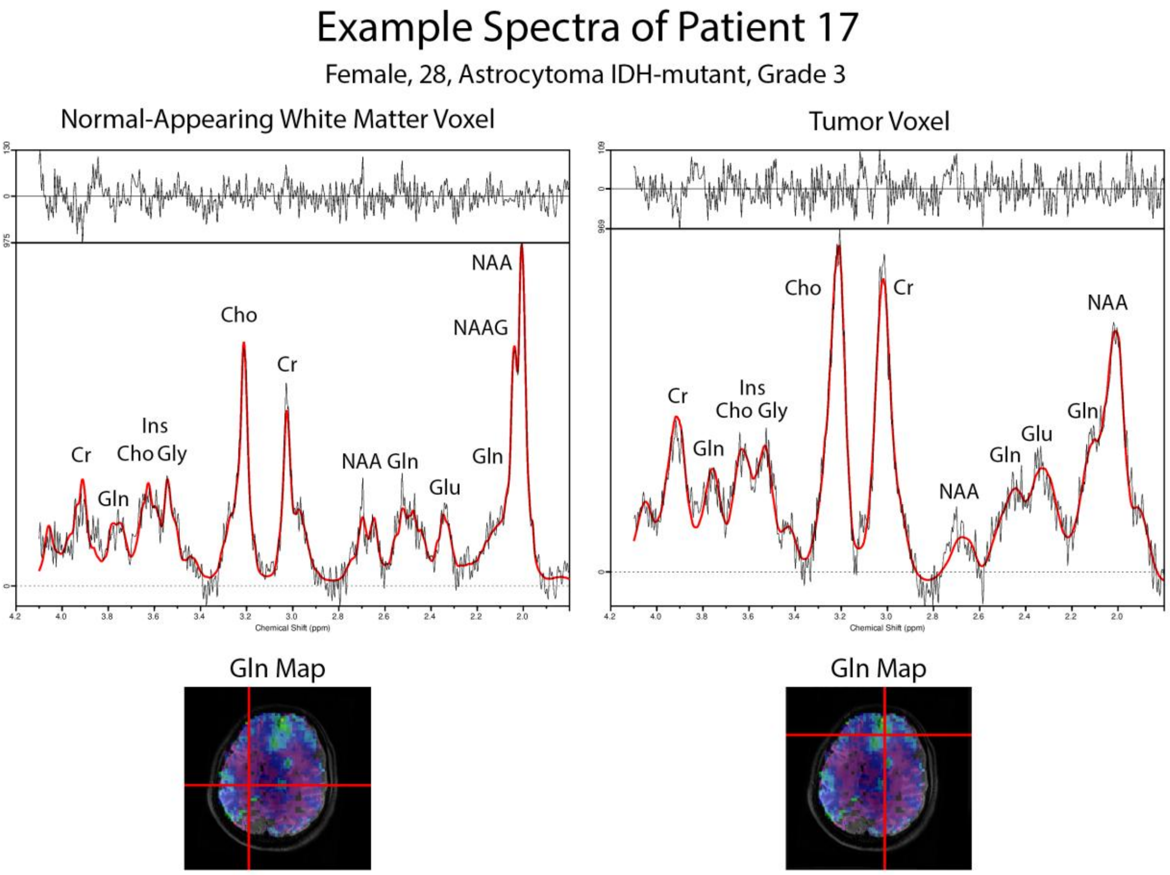


**Supplementary Figure 1**. Example spectra of patient 17 (astrocytoma IDH-mutant, grade 3, female, 28 years of age). Normal appearing white matter spectrum (left) and tumor spectrum (right). Below, glutamine (Gln) maps overlaid with a T1w reference image are shown, and the voxel position is indicated.

**Supplementary Table 1.** An overview according to the minimum reporting standards in MR spectroscopy[5].

| Minimum Reporting Standards in MR Spectroscopy - Overview | |
|---|---|
| **Site** | Vienna High Field MR Center |
| **1. Hardware** | |
| **a. Field strength** | 7T |
| **b. Manufacturer** | Siemens |
| **c. Model** | Magnetom |
| **d. RF coils: nuclei (transmit/ receive), number of channels, type, body part** | 1H, 32 ch, head, Nova Medical |
| **e. Additional hardware** | N/A |
| **2. Acquisition** | |
| **a. Pulse sequence** | FID-MRSI |
| **b. Volume of interest (VOI) locations** | Tumor |
| **c. Nominal VOI size** | 220×220×110 mm³ |
| **d. Repetition time (TR), echo time (TE)** | 450 ms / 1.3 ms acquisition delay |
| **e. Total number of excitations or acquisitions per spectrum** | N/A, spatial-spectral encoding |
| **In-time series for kinetic studies** | N/A |
| **i. Number of averaged spectra (NA) per time-point** | N/A |
| **ii. Averaging method (e.g., block-wise or moving average)** | N/A |
| **iii. Total number of spectra (acquired / in-time series)** | N/A |
| **f. Additional sequence parameters (spectral width in Hz, number of spectral points, frequency offsets); If STEAM: Mixing Time TM; If MRSI: 2D or 3D, FOV in all directions, matrix size, acceleration factors, sampling method** | BW 2778 Hz, 1920 spectral points, MRSI: 3D, 220×220×133 mm³, 64×64×39, spatial-spectral encoding |
| **g. Water suppression method** | WET |
| **h. Shimming method, reference peak, and thresholds for "acceptance of shim" chosen** | Standard shim + manual adjustment, water peak < 50 Hz |
| **i. Triggering or motion correction method** | N/A |
| **3. Data analysis methods and outputs** | |
| **a. Analysis software** | LCModel 6.3-1 |
| **b. Processing steps deviating from quoted reference or product** | N/A |
| **c. Output measure** | Ratio |
| **d. Quantification references and assumptions, fitting model assumptions** | Simulated in NMRScope-B, macromolecular background |
| **4. Data Quality** | |
| **a. Reported variables (SNR, linewidth (with ref. peaks))** | SNR and linewidths not reported |
| **b. Data exclusion criteria** | tCr SNR <5; tCr FWHM >0.15 ppm; metabolite Cramér-Rao lower bounds (CRLB) >40 % |
| **c. Quality measures of post processing model fitting** | CRLB |
| **d. Sample spectrum** | See Supp. Fig. 1 |

| This study | Hangel 2020 | Hangel 2022 | Lazen 2024 | Cadrien 2024 |
|---|---|---|---|---|
| 1 | | | | 1 |
| 2 | 1 | 1 | | 2 |
| 3 | 2 | | | 3 |
| 4 | 3 | | 1 | 4 |
| 5 | 4 | 2 | 2 | 5 |
| 6 | 5 | 3 | 3 | 6 |
| 7 | 6 | 4 | 4 | 7 |
| 8 | | 5 | 5 | 8 |
| 9 | | 6 | 7 | 10 |
| 10 | 9 | | | 12 |
| 11 | 11 | 7 | | |
| 12 | 12 | 8 | 8 | 13 |
| 13 | 13 | 9 | | 14 |
| 14 | 14 | 10 | | 15 |
| 15 | 16 | 12 | | 17 |
| 16 | 17 | | 9 | 18 |
| 17 | 20 | 13 | 10 | 20 |
| 18 | 21 | | | 21 |
| 19 | | 14 | | 22 |
| 20 | | 15 | 11 | 23 |
| 21 | | 16 | 12 | 24 |
| 22 | | | | |
| 23 | | 20 | | 30 |
| 24 | | | | |
| 25 | | | | |
| 26 | | 26 | | 35 |

**Supplementary Table 2.** Cohort overlap with previous publications[1–4].

**References:**


1. Hangel G, Cadrien C, Lazen P, et al. High-resolution metabolic imaging of high-grade gliomas using 7T-CRT-FID-MRSI. *NeuroImage Clin*. 2020;28:102433. doi:10.1016/j.nicl.2020.102433

2. Hangel G, Lazen P, Sharma S, et al. 7T HR FID-MRSI Compared to Amino Acid PET: Glutamine and Glycine as Promising Biomarkers in Brain Tumors. *Cancers*. 2022;14(9):2163. doi:10.3390/cancers14092163

3. Lazen P, Lima Cardoso P, Sharma S, et al. A Comparison of 7 Tesla MR Spectroscopic Imaging and 3 Tesla MR Fingerprinting for Tumor Localization in Glioma Patients. *Cancers*. 2024;16(5):943. doi:10.3390/cancers16050943

4. Cadrien C, Sharma S, Lazen P, et al. 7 Tesla magnetic resonance spectroscopic imaging predicting IDH status and glioma grading. *Cancer Imaging*. 2024;24(1):67. doi:10.1186/s40644-024-00704-9

5. Lin A, Andronesi O, Bogner W, et al. Minimum Reporting Standards for in vivo Magnetic Resonance Spectroscopy (MRSinMRS): Experts' consensus recommendations. *NMR Biomed*. 2021;34(5):e4484. doi:10.1002/nbm.4484
